# Prognostic prediction of glioblastoma by quantitative assessment of the methylation status of the entire *MGMT* promoter region

**DOI:** 10.1186/1471-2407-14-641

**Published:** 2014-08-30

**Authors:** Manabu Kanemoto, Mitsuaki Shirahata, Akiyo Nakauma, Katsumi Nakanishi, Kazuya Taniguchi, Yoji Kukita, Yoshiki Arakawa, Susumu Miyamoto, Kikuya Kato

**Affiliations:** Research Institute, Osaka Medical Center for Cancer and Cardiovascular Diseases, 1-3-3 Nakamichi, Higashinari-ku, Osaka, Japan; Department of Neurosurgery, Kyoto University Graduate School of Medicine, 54 Kawahara-cho, Shogoin, Sakyo-ku, Kyoto-shi, Kyoto, 606-8507 Japan; Department of Neuro-Oncology/Neurosurgery, Saitama Medical University International Medical Center, 1397-1 Yamane, Hidaka, Saitama, 350-1298 Japan

**Keywords:** Glioma, O6-methylguanine-DNA methyltransferase, Methylation, Bisulfite genome sequencing, Next-generation sequencing

## Abstract

**Background:**

O6-methylguanine-DNA methyltransferase (*MGMT*) promoter methylation is reported to be a prognostic and predictive factor of alkylating chemotherapy for glioblastoma patients. Methylation specific PCR (MSP) has been most commonly used when the methylation status of *MGMT* is assessed. However, technical obstacles have hampered the implementation of MSP-based diagnostic tests. We quantitatively analyzed the methylation status of the entire *MGMT* promoter region and applied this information for prognostic prediction using sequencing technology.

**Methods:**

Between 1998 and 2012, the genomic DNA of 85 tumor samples from newly diagnosed glioblastoma patients was subjected to bisulfite treatment and subdivided into a training set, consisting of fifty-three samples, and a test set, consisting of thirty-two samples. The training set was analyzed by deep Sanger sequencing with a sequencing coverage of up to 96 clones per sample. This analysis quantitatively revealed the degree of methylation of each cytidine phosphate guanosine (CpG) site. Based on these data, we constructed a prognostic prediction system for glioblastoma patients using a supervised learning method. We then validated this prediction system by deep sequencing with a next-generation sequencer using a test set of 32 samples.

**Results:**

The methylation status of the *MGMT* promoter was correlated with progression-free survival (PFS) in our patient population in the training set. The degree of correlation differed among the CpG sites. Using the data from the top twenty CpG sites, we constructed a prediction system for overall survival (OS) and PFS. The system successfully classified patients into good and poor prognosis groups in both the training set (OS, p = 0.0381; PFS, p = 0.00122) and the test set (OS, p = 0.0476; PFS, p = 0.0376). Conventional MSP could not predict the prognosis in either of our sets. (training set: OS; p = 0.993 PFS; p = 0.113, test set: OS; p = 0.326 PFS; p = 0.342).

**Conclusions:**

The prognostic ability of our prediction system using sequencing data was better than that of methylation-specific PCR (MSP). Advances in sequencing technologies will make this approach a plausible option for diagnoses based on *MGMT* promotor methylation.

**Electronic supplementary material:**

The online version of this article (doi:10.1186/1471-2407-14-641) contains supplementary material, which is available to authorized users.

## Background

A glioblastoma (GB) is a malignant brain tumor with a poor prognosis; the median survival time of GB patients is less than 2 years [1]. The current standard of care for GB patients is maximum surgical resection combined with radiation and concomitant adjuvant temozolomide (TMZ) therapy [2]. The long-term results of the EORTC-NCIC CE.3 trial revealed that the 5-year survival of GB patients approaches 10%, despite the largely poor prognosis [3]. Although novel drugs, such as molecular-targeted drugs, have been developed, their survival benefit has not been confirmed, and these molecular targeted drugs are known to carry risks of specific adverse events [4–6]. Accordingly, it is important to identify patients who may respond to conventional chemo-radiation therapy as part of future personalized care. Although nitrosoureas were commonly used for chemotherapy, TMZ is now used for first-line therapy. These drugs are alkylating agents that add an alkyl group to the O6 position of guanine, damaging the genomic DNA of cancer cells. O6-methylguanine-DNA methyltransferase (MGMT) removes alkyl groups from the O6 position of guanine and plays an important role in DNA repair [7–10]. Therefore, *MGMT* expression is associated with resistance to chemotherapeutic alkylating agents. The expression of *MGMT* is controlled by epigenetic gene silencing [11–13]. The methylation of the *MGMT* promoter is associated with sensitivity to alkylating chemotherapy drugs and is recognized as a prognostic factor for GB patients [14–18].

In recent years, TMZ monotherapy has been attempted for elderly GB or low-grade glioma patients, and an association between the treatment response and the *MGMT* methylation status has been examined [19, 20]. These studies demonstrated that the methylation status of *MGMT* is a strong predictive factor of TMZ monotherapy outcomes in elderly GB patients, and the clinical utility of the *MGMT* methylation status is increasing [21, 22].

Even with this accumulating clinical evidence, the implementation of diagnostic tests examining the methylation status of the *MGMT* promoter has been difficult. PCR-based techniques, such as methylation-specific PCR (MSP) and quantitative MSP, are the most popular methods of assessment [23, 24]. These techniques detect methylation sequences by sequence-specific binding of primers, which is an indirect method and only detects a limited number of methylation sites. DNA sequencing (i.e., bisulfite genomic sequencing) provides more direct information on methylation status. In this context, pyrosequencing is considered a good alternative. However, the target methylation sites of pyrosequencing are also limited [25, 26]. The *MGMT* promoter region spans more than one thousand base pairs and contains approximately one hundred potential methylation sites. To assess the methylation status of the *MGMT* promoter, it would be preferable to assess information from all methylation sites and select important CpG sites with survival analysis.

In this report, we performed deep sequencing of the *MGMT* promoter region after bisulfite treatment to clarify the global methylation status of the region. Because the methylation status is not uniform in glioma tissue, it is important to characterize the intratumor heterogeneity of *MGMT* promoter methylation. An analysis of survival data assessed the correlation between each CpG site and the malignancy of the glioblastoma. Based on this correlation, we built a classifier to predict the malignancy of GB using deep sequencing with a next-generation sequencer.

## Methods

### Patient characteristics

We obtained 85 GB specimens from patients who underwent surgical resection at Kyoto University Hospital and related regional hospitals between 1998 and 2012. The majority of the patients were recruited for a phase II clinical trial [27], and their tissues were used for studies on gene expression profiling [28, 29]. Histological diagnoses were established by the Kyoto University Pathology Unit according to the criteria established by the World Health Organization. The protocol was approved by the institutional review board of Kyoto University, and written informed consent was obtained from each of the patients. All tumor specimens were immediately snap frozen upon surgical resection and stored at -80°C until use. Tumor specimens containing 20% or more non-tumor tissue or necrotic areas were excluded from further analysis. The preoperative Karnofsky performance status score of each patient was at least 50 for each case. All patients received radiation therapy with and without alkylating chemotherapy postoperatively. The patient characteristics are shown in Table 1. We divided the data matrix into two data sets: one set consisted of 53 patients and was designated as the training set, and the other set contained 32 patients and was designated as the test set.

**Table 1:** **Patients’ clinical characteristics**

Sample		85	
Age		6-88	Median: 60
Gender	Female	36	
	Male	49	
Removal	Biopsy	1	
	Partial	29	
	Subtotal	28	
	Total	20	
	Unknown	8	
Post operative therapy	VAC-feron	57	
	Temozolomide	14	
	Other ACNU regimen	4	
	Radiation alone	7	
	Other	3	
Overall survival (months)		3-96	Median: 12
Progression free survival (months)		1-96	Median: 6

### DNA extraction and bisulfite treatment

Genomic DNA was extracted with the QIAamp DNA Mini Kit (Qiagen) according to the manufacturer’s instructions. One nanogram of genomic DNA was subjected to bisulfite treatment using the MethylEasy DNA Bisulfite Modification Kit (Takara) in accordance with the manufacturer’s instructions. We determined the quality of bisulfite-treated genomic DNA by real-time PCR of the actin gene as previously described [30]. The outline of the procedure is schematically shown in Additional file 1: Figure S1.

### Methylation-specific PCR (MSP)

Conventional MSP was performed as previously described [31]. PCR was performed using AmpliTaq Gold polymerase and the GeneAmp PCR system 9700 (Applied Biosystems). The sequences of the primer pairs were 5′-TTTGTGTTTTGATGTTTGTAGGTTTTTGT-3′ and 5′-AACTCCACACTCTTCCAAAAACAAAACA-3′ for unmethylated *MGMT* (fragment size: 93 bp) and 5′- TTTCGACGTTCGTAGGTTTTCGC -3′ and 5′-GCACTCTTCCGAAAACGAAACG-3′ for methylated *MGMT* (fragment size: 81 bp). These sequences and the PCR primer sequences used in the further analysis were constructed according to the *MGMT* promoter sequence (http://www.ncbi.nlm.nih.gov/nuccore/X61657.1). After an initial incubation at 95°C for 12 min, PCR amplification was performed with 40 cycles of 95°C for 15 sec, 59°C for 30 sec, and 72°C for 30 sec, followed by a 4-min final extension. The PCR products were electrophoresed on 2% agarose gels and were classified as methylated if a band with the PCR product was visualized using the methylated primer. The experiments were performed twice to confirm the reproducibility of the results. There were no discrepancies between duplicate reactions.

### Quantitative bisulfite genome sequencing (qBGS) of the training set

For qBGS, the *MGMT* promoter region was amplified by nested PCR. The sequences of the first-round PCR primers were 5′-TGGTAAATTAAGGTATAGAGTTTTAGG-3′ and 5′-GGTTAGGTGTTAGTGATGTT-3′. The PCR protocol was optimized for bisulfite-treated genomic DNA; each 10-μl reaction mixture of the modified protocol contained 2.5 mM MgCl_2_, 3% DMSO, 20 ng bisulfite-treated genomic DNA, and 1 μl of AmpliTaq Gold. After an initial incubation at 95°C for 12 min, PCR amplification was performed using 30 cycles of 95°C for 15 sec, 54°C for 30 sec and 72°C for 1 min, followed by a 4-min final extension. A 1-μl aliquot of the first-round PCR product was used as the template of the second-round PCR reaction. The sequences of the second-round PCR primers were 5′-TGGTAAATTAAGGTATAGAGTTTTAGG-3′ and 5′-TTGGATTAGGTTTTTGGGGTT-3′ (fragment size: 662 bp). The genomic position is chr 10: 131,155,100-131,155,761. The second-round PCR was performed using KOD-plus DNA polymerase (TOYOBO) according to the manufacturer’s instructions with 1.5 mM MgSO_4_ and 3% DMSO. After an initial incubation at 95°C for 2 min, PCR amplification was performed with 30 cycles of 94°C for 15 sec, 58°C for 30 sec, and 68°C for 1 min. The PCR products were purified using the MinElute PCR Purification Kit (QIAGEN) and ligated into the pCR-Blunt plasmid using the Zero Blunt PCR Cloning Kit (Invitrogen) and a DNA ligation kit (Takara). MAX Efficiency DH5 Competent Cells (Invitrogen) were used for transformations. A total of 96 colonies of each sample were subjected to bisulfite sequencing using a 3730xl DNA Analyzer (Applied Biosystems). The methylation status was analyzed with QUMA web tools (http://quma.cdb.riken.jp/).

### qBGS for the test set

For the test set, we used next-generation sequencing (MiSeq, Illumina) instead of Sanger sequencing. The target sequence was amplified by nested PCR. PCR amplification was performed using 40 cycles of 94°C for 30 sec, 54°C for 30 sec, and 72°C for 45 min, followed by a 4-min final extension. The sequences of the first-round PCR primers were 5′-GGATATGTTGGGATAGTT-3′ and 5′-CCAAAAACCCCAAACCC-3′ [26]. The sequences of the second-round PCR primers were 5′-GGATATGTTGGGATAGTT-3′ and 5′- AAATAAATAAAAATCAAAAC-3′ (fragment size: 216 bp). The annealing temperature was 48°C in the second-round PCR. The PCR product was attached with an adapter for MiSeq plus, consisting of an eight- or six-base index. The pooled PCR library of the test set samples was sequenced by paired-end sequencing with a MiSeq sequencer. Paired-end reads were aligned to a C-to-T converted reference sequence of the *MGMT* promoter region using BWA [32]. We used SAMtools to obtain the per-base coverage (pileup files) and counted non-bisulfite converted sites [33].

### Statistical analysis

Statistical analyses were performed using the free statistics software R (http://www.r-project.org/). Overall survival (OS) and progression-free survival (PFS) were defined as the period from surgery to death and from surgery to radiological detection of tumor progression, respectively. Tumor progression was diagnosed based on the criteria of the Brain Tumor Registry committee (Japan), which includes: a 25% increase in tumor size, the appearance of new lesions, or the obvious deterioration of the patient due to a mass effect or perifocal edema (in Table 1).

## Results

### Quantitative bisulfite genome sequencing of the training set

**Figure 1:**
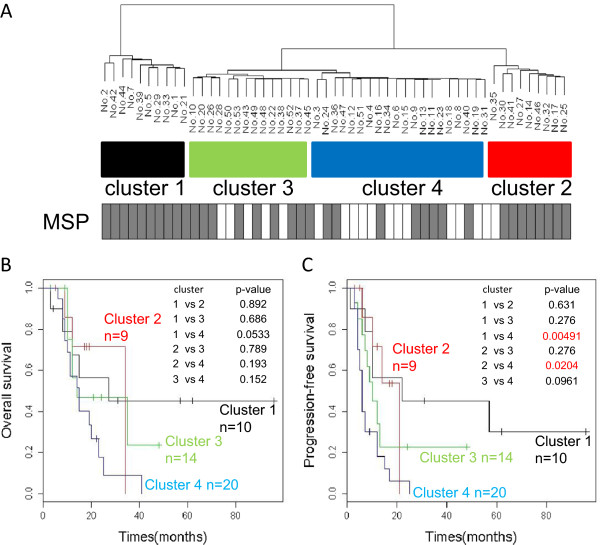
**Clustering of the training set and survival analysis.** Unsupervised analysis based on the *MGMT* methylation patterns. **(A)** A hierarchical cluster analysis of the methylation of the MGMT promoter in 53 samples. Cluster 1 (Black), strongly methylated samples; cluster 2 (red), moderately methylated; cluster 3 (green), slightly methylated; cluster 4 (blue), mostly unmethylated. The columns below the clustering show the results obtained using MSP. The gray column indicates methylated, and the white column is unmethylated samples. **(B, C)** Survival analysis was performed between all combinations of the four cluster subgroups. For PFS, the analysis showed statistically significant differences between cluster 1 and cluster 4 (p = 0.00491) and between cluster 2 and cluster 4 (p = 0.0204). For OS, there was no statistically significant difference between any combination of the four clusters, but there was a trend toward a difference between cluster 1 and cluster 4 (p = 0.0533).

**Figure 2:**
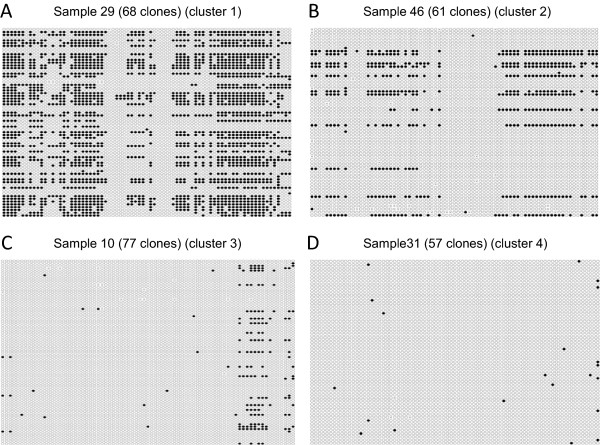
**Methylation pattern obtained by qBGS.** Methylation pattern observed using qBGS. The black and white circles indicate methylated and unmethylated CpG sites, respectively. Horizontally, 77 CpG sites are aligned. Vertically, the sequencing results of individual clones are aligned. **(A)** Sample 29 from cluster 1 of Figure 1; **(B)** sample 46 from cluster 2; **(C)** sample 10 from cluster 3; and **(D)** sample 31 from cluster 4.

Bisulfite sequencing was performed to fully analyze the methylation status of the *MGMT* promoter region. Due to intratumor heterogeneity, the methylation status of individual cells is not identical, even within a single glioma tissue. To clarify this heterogeneity, we performed quantitative bisulfite sequencing and obtained data from 25 to 81 molecules (median, 51) from each sample. This approach is referred to as quantitative bisulfite genome sequencing (qBGS). The 662-bp fragment subjected to qBGS contained 78 CpG sites. One CpG site that is not located within the CpG island of the *MGMT* promoter region was excluded from further analysis. The methylation proportion at each CpG site was calculated as the fraction of clones with a methylated C at that site in all sequenced clones. The methylation status of the *MGMT* promoter region was then described as a data point in a 77-dimensional space constructed from the methylation proportions of the 77 CpG sites. We performed a hierarchical cluster analysis with the Ward method using the raw methylation proportion without any standardization to obtain a general view of the global methylation features of the *MGMT* promoter region. The cases were grouped into four clusters (Figure 1A). These clusters were correlated with the degree of methylation. The column bars below the clustering indicate the MSP results for 53 samples. Typical examples of qBGS results are shown in Figure 2. The samples in cluster 1 were strongly methylated, the samples in cluster 2 were moderately methylated, the samples in cluster 3 were slightly methylated, and the samples in cluster 4 were almost unmethylated. There was a trend toward a prognostic difference for OS between cluster 1 and cluster 4 (p = 0.0533) (Figure 1B). Statistically significant associations with PFS were observed between clusters 1 and 4 (p = 0.00491) and between clusters 2 and 4 (p = 0.0204) (Figure 1C). Several cases that were judged to be methylated (i.e., to have a good prognosis) by MSP belonged to clusters 3 and 4 (Figure 1A). For example, samples 13 and 16 belonged to cluster 4; both showed four months of PFS and were described as poor prognosis [2], but were judged to be methylated and to have a good prognosis by MSP.

**Figure 3:**
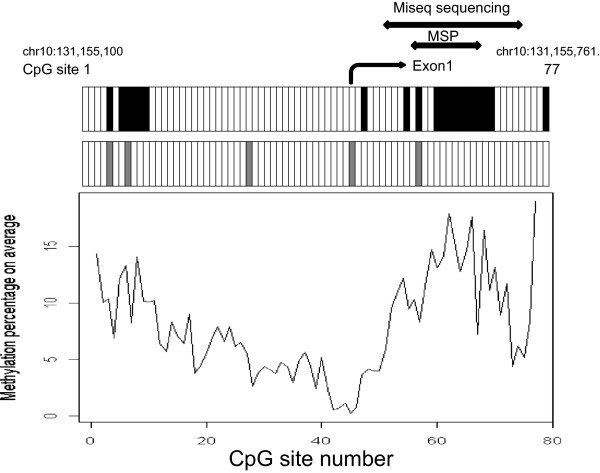
**Proportion of methylation status and survival analysis at each CpG site.** Average of the methylation percentage of CpG sites. The black and gray columns in the top panel indicate CpG sites with correlations with PFS and OS, respectively, that exceed the threshold (p < 0.05).

To demonstrate an overview of the methylation status of the *MGMT* promoter region, the averages of the methylation proportions of the CpG sites are shown in Figure 3. The promoter sequence may be divided into three segments according to the methylation proportions. The methylation level of the CpG sites in the middle segment, from CpG28 to CpG50, was lower than that of the other segments (Figure 3). This area is located just upstream of the transcription start site. We performed univariate Cox proportional hazard analysis of PFS to identify prognostically important CpG sites using the methylation proportion as a continuous variable. Based on an analysis using the 53 training samples, the log-rank p values of 20 CpG sites were less than 0.05. These 20 selected CpG sites were CpG63 (p = 0.0056), CpG64 (p = 0.0088), CpG77 (p = 0.010), CpG62 (p = 0.012), CpG56 (p = 0.012), CpG68 (p = 0.014), CpG11 (p = 0.023), CpG65 (p = 0.025), CpG66 (p = 0.025), CpG59 (p = 0.027), CpG8 (p = 0.028), CpG60 (p = 0.028), CpG10 (p = 0.030), CpG7 (p = 0.034),CpG5 (p = 0.034), CpG61 (p = 0.035), CpG54 (p = 0.038), CpG9 (p = 0.038), CpG47 (p = 0.047), and CpG67 (p = 0.048). Almost all of the selected sites were located at positions from CpG5 to CpG11 or from CpG54 to CpG68 (black columns in Figure 3). However, only five CpG sites were selected for OS under the same condition: CpG8 (p = 0.039), CpG28 (p = 0.041), CpG56 (p = 0.041), CpG5 (p = 0.044), and CpG45 (p = 0.049) (gray columns in Figure 3). Three CpG sites, CpG5, CpG8, and CpG56, showed a correlation with OS and PFS. All of the results of univariate Cox analysis are supplied in Additional file 2 (PFS) and Additional file 3 (OS). Shah et al. reported a similar comprehensive methylation analysis [34]. Their numbering scheme of CpG sites corresponds to the addition of twenty to our numbering scheme of sites.

### Diagnostic system for prognosis prediction using quantitative methylation data

**Figure 4:**
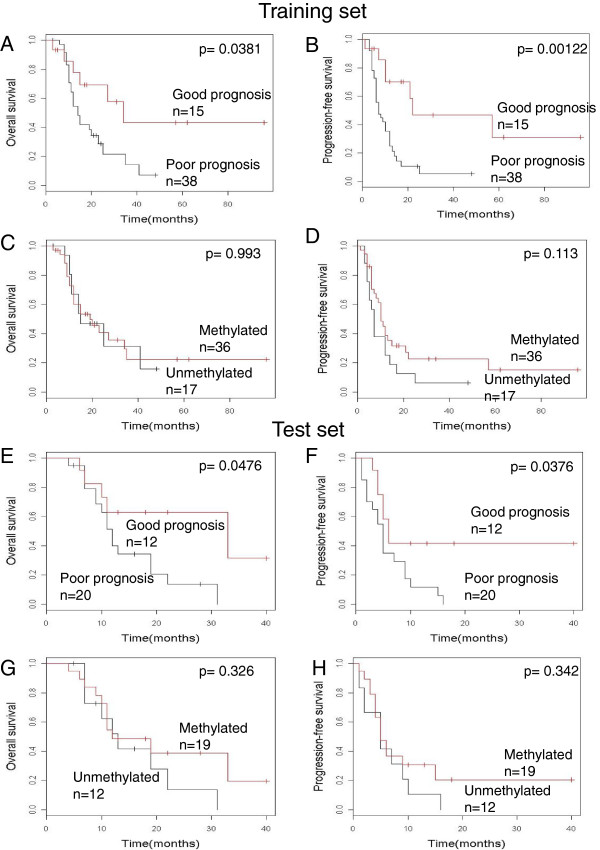
**Survival analysis of the training set by M-score and MSP.** In each panel, the red line indicates either a good prognosis (M-score) or the methylated (MSP) group. The black line indicates either a poor prognosis (M-score) or the unmethylated (MSP) group. **(A)** training set, M-score, OS. **(B)** training set, M-score, PFS. **(C)** training set, MSP, OS. **(D)** training set, MSP, PFS. **(E)** test set, M-score, OS. **(F)** test set, M-score, PFS. **(G)** test set, MSP, OS. **(H)** test set, MSP, PFS.

As described above, the prognostic significance of each CpG site is limited, and it would be more effective to combine the information from multiple CpG sites. One approach is an unsupervised analysis, including a cluster analysis, shown above. However, to construct a diagnostic system, supervised learning is more appropriate. Here, based on the correlation between OS or PFS and the methylation status of the *MGMT* promoter region, we constructed a diagnostic system to predict the therapeutic outcomes of GB patients based on the methylation proportion of CpG51 - CpG74. Because we intended to use a next-generation sequencer for the validation study, we selected the CpG sites to be examined based on the read length restriction of the sequencer. This diagnostic score was denoted as the M-score (methylation score) and is defined as a weighted sum of the methylation proportion as follows:


where ‘*A*_*i*_’ is a regression coefficient deduced by univariate Cox analysis of PFS at CpG site *i* and ‘*X*_*i*_’ is the methylation proportion at CpG site *i*. As described above, a correlation between OS and the methylation status was not clear in our patient population. We therefore used the same M-score calculation formula for OS as well. First, the performance of the M-score diagnostic system was evaluated by leave-one-out-cross-validation (LOOCV) using the 53 training samples. The 53 samples were divided into groups consisting of one and 52 samples, and *‘Ai’* was calculated by univariate Cox analysis using the data for the remaining 52 samples. The threshold was selected from M-scores of the 52 samples so that the log-rank p value of the Kaplan-Meier analysis for the two divided groups was minimized. In cases of multiple M-scores with the same minimum p value, the median was selected as the threshold. Next, the M-score of the one sample was calculated using parameters deduced from the 52 samples, and the sample was classified into either the good or poor prognosis group using the threshold. This process was repeated until all samples were tested. The LOOCV procedure is schematically shown in Additional file 1: Figure S2. The results of the LOOCV procedure are shown in Figure 4A and B; this approach demonstrated excellent prognostic ability with OS and PFS (OS, p = 0.0381; PFS, p = 0.00122). Thus, the diagnostic accuracy of our system is better than that of the MSP-based approach (Figure 4C, D) (OS, p = 0.993; PFS, p = 0.113).

### Validation of the diagnostic system using next-generation sequencing

For validation of the test set, the parameters (*Ai*) were calculated using all 53 samples in the training set, and the threshold was set at 2.2, the average of the thresholds of the 53 LOOCV processes.For the 32 test set samples, we performed qBGS with a next-generation sequencer, MiSeq, to examine the potential future applications of this approach. We also performed MSP in all cases except one, due to the loss of genomic DNA. The mean depth of MiSeq sequencing was 80,817 reads. The methylation proportion of each CpG site was obtained, M-scores were calculated, and the test set samples were classified using the threshold listed above. Survival analysis indicated a statistically significant difference between the two groups with respect to PFS (p = 0.0376) and OS (p = 0.0476) (Figure 4E, F). There was no statistically significant difference between the two groups by classification with MSP (OS, p = 0.326; PFS, p = 0.342) (Figure 4G, H).

For potential future applications of this technique, we designed PCR primers that amplify the same region from FFPE samples. The method and results are shown in Additional file 4.

### Multivariate Cox regression analysis

We performed Cox regression analysis to evaluate clinical parameters, such as age (above or below 60), gender, the extent of resection, post-operative chemotherapy (VAC-feron or TMZ), and the methylation status by the M-score sequencing method as predictors of OS and PFS in the GB patients in the test set. The variables with a p value < 0.2 were analyzed with a backward stepwise Multivariate Cox proportional hazard model. For OS, the best predictor was the M-score (p = 0.0585) (Hazard Ratio, 0.3558), and the next best prognostic factor was the extent of surgical resection (p = 0.0739) (Hazard Ratio, 0.5996). The M-score was found to be the best predictor of PFS (p = 0.0247; Hazard Ratio, 0.334).

## Discussion

In this report, we characterized the methylation status of the entire *MGMT* promoter region using deep sequencing. The methylation status of each CpG site was quantitatively evaluated by sequencing multiple clones. Based on these results, we constructed a prognosis predictor that incorporates the methylation status of multiple CpG sites using supervised learning. The construction of a classifier using supervised learning is popular in the field of gene expression profiling, and we demonstrated here that the same approach is effective for the prediction of methylation status.

In our patient population, the correlation of the methylation status with OS was less clear than that with PFS. This is most likely due to variation of the therapy used after the first line therapy. The majority of our patients received repeated surgical resections, second line chemotherapy or additional radiotherapy. For multivariate analysis, age was not a prognosis factor, unlike in the past reports. We also performed surgical medical treatment with methylation-positive elderly patients. In particular, repeated surgery was likely to prolong the survival time of the glioblastoma patients with a poor prognosis.

MSP is the most widely used assay for methylation. However, MSP can only detect the CpG sites in the primer region; the methylation status of other CpG sites has no effect on the amplification. In a prior study, only 12.5% of the results obtained from two MSP experiments matched when the forward and reverse primers were different [35]. In addition, there is no established method to confirm the quality of bisulfite-converted genomic DNA. We assessed the quality using the Ct value of actin in real-time PCR. Approximately 64% of our glioma samples were methylation-positive with MSP. The positive rate was higher than that in other studies with some exceptions [36, 37]. We excluded samples damaged by bisulfite treatment in the actin-based confirmation system, and this process may have increased the positive rate. This discrepancy in MSP results, which is most likely a false positive, might be influenced by the T genotype of the *MGMT* C > T (rs16906252) enhancer single-nucleotide polymorphism (SNP), which was reported by McDonald et al. [38] to interact with MGMT promotor methylation. Vlassenbroeck et al. also evaluated the results of qMSP based on the copy number of actin using real-time PCR [39]. It is often difficult to set a threshold for agarose gel patterns of MSP. This problem has been overcome by quantitative MSP [40, 41]. Quantitative MSP was applied in two recent phase 3 trials of glioma [21, 22]. However, the problem of limited coverage of CpG sites by MSP remains in need of technical improvements.

As discussed above, bisulfite sequencing can cover all CpG sites. In this context, pyrosequencing is considered to cover more CpG sites than MSP [26]. The methylation proportions can be semi-quantitatively deduced from the peak height of each incorporated nucleotide. The main disadvantage of pyrosequencing is its short read length [25, 26]. qBGS using Sanger sequencing is not subject to this limitation, and its moderate read depth provides more accurate quantitative information. Because deep sequencing with the Sanger method is laborious, the use of next-generation sequencing may make this approach more comparable to pyrosequencing.

The major shortcoming of qBGS and pyrosequencing is the absence of a consensus regarding the data handling of multidimensional quantitative data. Dunn et al. and Motomura et al. used the average of the methylation proportion of multiple CpG sites (CpG51 - CpG62, Dunn et al.; CpG2 - CpG16, Motomura et al.) [42, 43]. Karayan-Tapon et al. used the methylation proportion of five CpG sites (CpG 53–57) and grouped patients using the median value of the methylation proportion as the threshold [25]. We developed the M-score diagnostic system using the analysis method of gene expression profiling and calculated the optimized threshold by LOOCV. The M-score is the weighted sum of the methylation proportions of multiple CpG sites, which maximizes the correlation with the survival time. Our approach is more advanced than a simple summation of the population of methylated sites, and adding data from a larger patient population will improve the performance of the predictor. Bady et al. examined the quantitative value of 18 CpG sites in the *MGMT* promoter area using the Infinium methylation BeadChip and revealed two distinct CpG sites (CpG10 and CpG68). They converted multidimensional data to one methylation probability score using the inverse logit function. The classifier was validated with an external data set [44]. Both studies indicate a new direction for *MGMT* methylation assays based on evaluation of multiple CpG sites.

Shah et al. also quantitatively evaluated the methylation of the *MGMT* promoter [34]. Although the number of sequenced clones in that study was far less than that of our study (median of 10 clones), their results were similar to our results; the CpG sites located downstream of the transcription start site were often correlated with PFS. This prior study indicates that our observations are likely to be universal, and suggests that our prognosis predictor may be applicable to other patient populations.

The identification of biomarkers of gliomas has been an active area of research in recent years. It is well known that *IDH* mutations are a strong prognostic factor [45]. *IDH* mutations are associated with a hypermethylation phenotype [46], suggesting that the methylation of the *MGMT* promoter is one part of a genome-wide methylation profile [47]. Based on qBGS analysis, we identified different extents of methylation of CpG sites in the *MGMT* promoter region.

Recently, the methylation status of *MGMT* has become a focal point in the management of elderly GB patients. Two *MGMT* methylation analyses using samples from large phase 3 trials with elderly GB patients demonstrated that TMZ monotherapy was superior to conventional radiation therapy for the management of *MGMT*-methylated GB patients. Conversely, TMZ monotherapy was inferior to radiation therapy in GB cases with unmethylated *MGMT* [21, 22]. These results indicate that the *MGMT* methylation status is a strong predictive factor for the efficacy of TMZ monotherapy in elderly GB patients and that evaluating *MGMT* methylation status is necessary for the management of these patients. The relationship between the efficacy of TMZ monotherapy and qBGS-based methylation analysis of the *MGMT* promoter in elderly GB merits further investigation.

In addition to its application for elderly patients, TMZ monotherapy has been utilized for low-grade glioma patients [20, 48]. In this group, the co-deletion of 1p19q and *IDH* mutations were molecular prognostic factors. Given the findings in elderly GB patients, the methylation status of the *MGMT* promoter may also predict the outcomes of low-grade glioma patients treated by TMZ monotherapy. Because the *MGMT* promoter in normal tissue is generally unmethylated, methylated *MGMT* cases are susceptible to contamination by normal tissue. An advantage of qBGS is that it is easy to observe the state of contamination. qBGS also revealed intratumoral heterogeneity in the methylation of the *MGMT* promoter, which should be considered when using other methylation assays. Although qBGS is complicated and time-consuming, it is an important process for evaluating the methylation features of the *MGMT* promoter.

## Conclusions

We constructed a novel diagnostic system to predict the prognosis of glioblastoma patients using information regarding the methylation status of the entire *MGMT* promoter region. A precise assessment of the methylation status of the *MGMT* promoter may improve the prediction of disease progression and assist in the choice of TMZ treatment.

## Electronic supplementary material

Additional file 1: Figure S1: Algorithm of quality assessment of bisulfite-treated genomic DNA. **Figure S2.** Schematic representation of leave-one-out cross-validation. (DOC 228 KB)

Additional file 2: Table S1: Table of regression coefficients of CpG sites based on PFS. (XLS 26 KB)

Additional file 3: Table S2: Table of regression coefficients of CpG sites based on OS. (XLS 34 KB)

Additional file 4: **Agarose gel image of PCR product using FFPE genomic DNA.** (DOC 338 KB)

## Abbreviations

MGMT: O6-methylguanine-DNA methyltransferase
MSP: Methylation specific PCR
CpG: cytidine phosphate guanosine
PFS: Progression-free survival
OS: Overall survival
GB: Glioblastoma
TMZ: Temozolomide
qBGS: Quantitative bisulfite genome sequencing
DMSO: Dimethyl sulfoxide.
