# Supplementary material for: Prognostic prediction of glioblastoma by quantitative assessment of the methylation status of the entire MGMT promoter region
Source: BMC Cancer. 2014 Aug 30;14:641. doi: 10.1186/1471-2407-14-641 (PMC4161852; doi:10.1186/1471-2407-14-641)
Supplement: Supplementary file 1 — Additional file 1: Figure S1: Algorithm of quality assessment of bisulfite-treated genomic DNA. Figure S2. Schematic representation of leave-one-out cross-validation. (DOC 228 KB) [file 12885_2014_4817_MOESM1_ESM.doc]

Figure S1. Quality assessment of bisulfite-treated genomic DNA

Figure S2. Schematic representation of leave-one-out cross-validation
