# Supplementary material for: Prognostic prediction of glioblastoma by quantitative assessment of the methylation status of the entire MGMT promoter region
Source: BMC Cancer. 2014 Aug 30;14:641. doi: 10.1186/1471-2407-14-641 (PMC4161852; doi:10.1186/1471-2407-14-641)
Supplement: Supplementary file 4 — Additional file 4: Agarose gel image of PCR product using FFPE genomic DNA. (DOC 338 KB) [file 12885_2014_4817_MOESM4_ESM.doc]

**Supplementary Methods**

**DNA extraction and bisulfite treatment**

FFPE genomic DNA was extracted with the QIAamp DNA Mini Kit (Qiagen) according to the manufacturer's instructions. Genomic DNA was subjected to bisulfite treatment using MethylEasy Xceed (Takara) in accordance with the manufacturer's instructions.

**PCR amplification**

The *MGMT* promoter sequence was amplified by two-part nested PCR. The sequences of first-round primers of MGMT1 were 5'-GGATATGTTGGGATAGTT-3’ and 5'-AACCAATAAAACCTACTCCTCCCTTAA-3’ (fragment size: 99 bp). The sequences of the second-round PCR primers were the same as those used in first-round PCR. The sequences of first-round primers of MGMT2 were 5'- ATTTGGTGAGTGTTTGGG-3’ and 5'-CTAAAACTCRCCCRAAAT-3’ (fragment size: 150 bp). The sequences of second-round primers of MGMT2 were 5'- ATTTGGTGAGTGTTTGGG-3’ and 5'-AAATAAATAAAAATCAAAAC-3’ (fragment size: 163 bp).　PCR amplification of MGMT1 and MGMT2 were performed under the same conditions. The annealing temperature was 52°C in the first-round and second-round PCRs, which were performed using EpiTaq™ HS (TaKaRa).

**Figure S3**. Agarose gel image of the PCR product using FFPE genomic DNA. A) Actin, B) First round PCR product of MGMT1, C) First round PCR product of MGMT2, D) Second round PCR product of MGMT1, E) Second round PCR product of MGMT2.
